# *ALK*-Rearranged Lung Cancer in Chinese: A Comprehensive Assessment of Clinicopathology, IHC, FISH and RT-PCR

**DOI:** 10.1371/journal.pone.0069016

**Published:** 2013-07-26

**Authors:** Yuan Li, Yunjian Pan, Rui Wang, Yihua Sun, Haichuan Hu, Xuxia Shen, Yongming Lu, Lei Shen, Xiongzeng Zhu, Haiquan Chen

**Affiliations:** 1 Department of Pathology, Fudan University Shanghai Cancer Center, Shanghai, China; 2 Department of Thoracic Surgery, Fudan University Shanghai Cancer Center, Shanghai, China; 3 Department of Oncology, Shanghai Medical College, Fudan University, Shanghai, China; UNIVERSITY MAGNA GRAECIA, Italy

## Abstract

Approximately 3–7% of non-small cell lung cancers harbor an anaplastic lymphoma kinase (*ALK*) gene fusion, constituting a new molecular subtype of lung cancer that responds to crizotinib, an *ALK* inhibitor. Although previous studies have evaluated *ALK*-rearranged lung cancers, the comprehensive analysis of lung cancer in Chinese has not well assessed. Herein, we identified 44 cases of *ALK*-rearranged samples by fluorescent in-situ hybridization (FISH), immunohistochemistry (IHC), and reverse transcription polymerase chain reaction (RT-PCR) in a large number of surgically resected lung cancers. All 44 *ALK*-rearranged lung cancers were adenocarcinomas, with 2 cases having additional focal squamous components. The goal was to analyse the clinicopathological features of *ALK*-rearranged lung adenocarcinomas. Our data showed that a cribriform structure, prominent extracellular mucus and any type of mucous cell pattern may be either sensitive or specific to predict an *ALK* rearrangement. We used FISH as the standard detection method. We compared the *ALK* rearrangement accuracy of FISH, RT-PCR and IHC. RT-PCR could define both the *ALK* fusion partner and the fusion variant, but seemed unable to detect all translocations involving the *ALK* gene. It is noteworthy that IHC using the D5F3 antibody (Cell Signaling Technology) showed higher sensitivity and specificity than the ALK1 antibody (Dako). Therefore, we conclude that IHC remains a cost-effective and efficient technique for diagnosing *ALK* rearrangements and that D5F3 can be the optimal screening antibody in clinical practice.

## Introduction

Lung cancer remains the leading cause of cancer-related death in the world. The clinical importance of the molecular phenotype of lung cancer lies mainly in its therapeutic implications since different subtypes may respond to different target treatments. A fusion gene that joins the echinoderm microtubule-associated protein-like 4 (*EML4*) gene with the anaplastic lymphoma kinase (*ALK*) gene was found in a subset of non-small-cell lung carcinomas (NSCLCs) in 2007 [1]. This fusion occurred due to chromosomal inversion or translocation on chromosome 2p, resulting in formation of the *EML4-ALK* fusion gene. Despite the relatively low frequency of the *EML4-ALK* fusion, *ALK*-rearranged lung cancer is a unique molecular subgroup with a high sensitivity to ALK inhibitors, and is mutually exclusive from other well-known oncogenic mutations involving EGFR or KRAS [2]–[5]. To better understand *ALK*-rearranged lung cancers, it is important to characterize their clinicopathologic features. The results of detailed clinicopathologic analyses have varied. In our study, 572 lung adenocarcinomas were divided into *ALK*-rearranged, common driver mutation, and pan-negative groups according to mutational status. We compared clinicopathological features of the *ALK*-positive group with common driver mutation, and with pan-negative group, seeking to assess whether there were distinctive clinicopathological features associated with *ALK* rearrangement cohort.

*ALK*-rearranged lung cancer can be identified by IHC, FISH, or RT-PCR with each method having advantages and disadvantages for screening. Current clinical trials of crizotinib use FISH as the diagnostic test [6]. However, FISH requires specialized technical resources and expertise, and the signals rapidly fade over time. Furthermore, the cost of FISH assay limits its application for screening in clinical practice. RT-PCR coupling direct sequencing has been used to identify known fusion variants, but may miss unknown variants. IHC is relatively inexpensive, faster, and performed routinely on formalin-fixed paraffin-embedded (FFPE) tissues. However, the results of ALK IHC testing in lung cancer remains to be unsatisfied because of relatively low sensitivity or specificity, and absence of an ideal antibody. Now, the highly sensitive ALK monoclonal antibody, D5F3 can recognize epitopes within the ALK protein of all known fusions. IHC is readily available in routine pathology practice and the test costs only one- twentieth of FISH test in China. Therefore, this IHC analysis holds the promise of being cost-effective and a rapid screening method [7]. However, the accuracy and sensitivity of the D5F3 antibody have not been thoroughly tested. We compared the concordance among IHC, FISH and RT-PCR to determine the optimal screening method. FISH is the most accurate method and RT-PCR can define most ALK fusion variants, but may not detect all ALK translocations. We found that IHC with D5F3 monoclonal antibody showed both high sensitivity and specificity (91% and 98%, respectively) and thus might be routinely applicable in clinical practice.

## Materials and Methods

### Case Selection

A total of 572 frozen lung adenocarcinoma tissue samples and their corresponding paraffin-embedded tumor tissues were obtained at the Fudan University Shanghai Cancer Centre from Oct 2007 through Jun 2011, with written informed consents from all patients. All research involving human participants in this study was approved by the Ethics Committee of Shanghai Cancer hospital (shanghai cancer center), Fudan University, China. We previously demonstrated that 90% of lung adenocarcinomas from never-smokers harbored mutually exclusive oncogenic mutations in just four genes (*EGFR*, *KRAS*, *HER2* and ALK) [8]. In current study, we had included 527 lung adenocarcinomas irrespective of smoking status besides the 45 tumors (41 *EGFR*, 3 *EML4-ALK* fusions and 1 *KRAS*) previously published [8]. There were 411 of 572 samples which harbored known mutations, including 361 *EGFR*, 40 *KRAS* and 10 *BRAF* tumors, while 161 of 572 samples did not harbor any of the above mutations. For detection of *EML4-ALK* fusions, primers were designed to amplify all known fusion variants, using cDNA, as described previously [9]. According to mutational status, 572 lung adenocarcinomas were divided into three groups. There were 44 cases identified as ALK rearrangement by FISH and grouped as *ALK*-positive. There were 411 cases which harbored well-known oncogenic mutations (361 *EGFR*, 40 *KRAS* and 10 *BRAF*) identified by RT-PCR and grouped as common drive mutations. There were 117 cases which did not harbor any of the above mutation and were thus grouped as pan-negative. Clinicopathological data collected for analyses included age, sex, tumor size, smoking history, pleural invasion, lymph node status and pathologic stage. For each case, multiple slides corresponding to whole tissue sections were reviewed by two pathologists and classified according to the current IASLC/ATS/ERS classification [10]. All the tumors were graded by pattern-based grading system proposed by Sica into grade I, grade II, or grade III. Grade I, a lepidic predominant pattern; Grade II, an acinar, papillary or mucinous predominant pattern; and Grade III, a solid, micropapillary or cribriform pattern [11]. Cases with differences between reviewers were reevaluated and a consensus interpretation was rendered.

### RT-PCR and Mutational Analyses

There were 572 cases subjected to RT-PCR. Frozen tissues were grossly dissected into TRIZOL (Invitrogen Inc.) for RNA extraction following standard protocols. Total RNA samples were reverse transcribed into single-stranded cDNA using RevertAidTM First Strand cDNA Synthesis Kit (Fermentas, EU). Mutational status of *EGFR* (exons 18–22), *KRAS* (exons 2 to 3) and *BRAF* (exons 11 to 15) was accessed with standard RT-PCR and coupled direct sequencing. For detection of *EML4-ALK* fusions, primers were designed to amplify all known fusion variants using cDNA, as described previously [8], [9]. Forward primers included *EML4* E2F (5′-TGATGTTTTGAGGCGTCTTG-3′), *EML4* E13F (5′-AGATCGCCTGTCAGCTCTTG-3′), *EML4* E18F (5′-TTAGCATTCTTGGGGAATGG-3′), and reverse primer *ALK* E20R (5′-TGCCAGCAAAGCAGTAGT TG-3′). Multiplex PCR analysis was done with KOD plus DNA polymerase (Toyobo, Osaka, Japan), with the following program: 94°C 5 minutes; 94°C 30 seconds, 60°C 30 seconds, 68°C 1 minute, 35 cycles; 68°C 10 minutes. PCR products were directly sequenced in forward and reverse directions. All mutations were verified by analysis of an independent PCR isolate. PCR products were directly sequenced in forward and reverse directions. All mutations were verified by analysis of an independent PCR isolate.

### Fluorescence in situ hybridization

*ALK*-rearranged lung cancer is a molecular subgroup and mutually exclusive from other mutations such as *EGFR*, *KRAS* and *BRAF*. The 161 cases which did not harbor any of the above mutations were subjected to FISH. FISH was performed on formalin-fixed paraffin-embedded (FFPE) specimens using a commercially available break-apart probe specific to the *ALK* gene (Vysis LSI ALK Dual Color, break-apart rearrangement probe; Abbott Molecular, Abbott Park, IL) according to manufacturer's instructions. The probe hybridized to band 2p23, on either side of the ALK gene breakpoint. The 5′ ALK signal was labeled with Spectrum Green (green), and the 3′ *ALK* signal was labeled with Spectrum Orange (orange). 4, 6-diamidino-2-phenylindole (DAPI) II was applied for nuclei counterstaining. Signals for each probe were evaluated under microscope equipped with triple-pass filter (DAPI/Green/Orange; Abbott Molecular) and oil immersion objective lens. FISH-positive cases were defined as two positive *ALK* rearrangement patterns. One was the break-apart pattern with one fusion signal and two separated orange and green signals. The distance between two separated signals was estimated using twice the size of the biggest signal size. Another definition was an isolated red signal pattern with one fusion signal and one red signal without a corresponding green signal. Positive cases were defined as more than 15% break-apart signals or isolated red signal signals in 50 tumor cells. FISH-negative cases were defined as those presenting overlapping of red and green signals (yellowish) in tumor cells, indicating that *ALK* was not rearranged.

### Immunohistochemistry

There were 161 cases which did not harbor any of above mutations. These were subjected to D5F3, ALK1, p63 and TTF-1 IHC. IHC was performed on 4 µm thick FFPE sections. Slides were deparaffinized and pretreated with 1 mmol/L EDTA and heat-mediated antigen retrieval solution in a microwave oven. Further steps were done at room temperature in a hydrated chamber. Slides were preincubated in 20% normal goat serum. ALK (D5F3) XP Rabbit monoclonal antibody (Cell Signaling Technology, Danvers,MA), Mouse monoclonal antihuman ALK1 (Dako) at 1∶10 dilution in Dako diluent, p63 (1∶400, 4A4, DAKO), and TTF-1 (1∶100, 8G7G3/1, DAKO) were applied. The slides were then washed in Tris-HCl and detected with horseradish peroxidase-conjugated anti-rabbit EnVision+ kit (Dako). All slides were counterstained with hematoxylin.

Cytoplasmic staining was regarded as positive for D5F3 and ALK1. This recognizes epitopes within the ALK protein that are preserved in all known ALK fusions. IHC staining was scored as follows: 0 (no staining); 1 (faint, cytoplasmic staining); 2 (moderate, smooth cytoplasmic staining); 3 (intense, granular cytoplasmic staining) in more than 10% of tumor cells. Scoring was performed by two pathologists. Nuclear staining of tumor cells was considered positive for TTF-1 and p63.

### Statistical analysis

The associations of lung adenocarcinomas with *ALK*-positive and common driver mutation or pan-negative group with clinical characteristics were assessed using Chi-square (for categorical data) and Wilcoxon rank-sum (for continuous data) tests. *p* values were based on a two-sided hypothesis. *p* values less than 0.05 were considered significant.

## Results

### Clinical features of *ALK*-rearranged lung cancer

The 572 surgically resected stage I–IV lung adenocarcinomas patients were all from China, their ages ranging from 22 to 84 years, with an average of 59 years. Patients were composed of 215 men and 357 women. Tumor diameters ranged from 0.5 to 11 cm. Pathologic stages were I in 285 and II–IV in 287 patients. According to mutational status, the 572 patients were divided into three groups, named *ALK*-positive group (44 cases), common driver mutation group (411 cases) and pan-negative group (117 cases). In all *ALK*-positive cases, no concurrent activating mutations in *EGFR*, *KRAS* and *BRAF* were detected. We compared clinicopathological features of the *ALK*-positive group with the common driver mutation and pan-negative groups. Of the cases with clinical data available, *ALK*-positive lung cancers were associated with younger age compared with the common driver mutation group (*p* = 0.023, Table 1), but age in *ALK*-positive group was not significantly difference from the pan-negative group (*p* = 0.83, Table 1). The *ALK*-positive group was associated with no-smoking history compared with pan-negative patient (*p*<0.001, Table 1), while the *ALK*-positive group showed no significance difference compared with the common driver mutation group (*p* = 0.828, Table 1). Although *ALK*-rearranged patients showed a tendency for association with positive lymph nodes status and higher stage in previous reports [12], [13], our *ALK*-positive group had no significance difference from the other 2 groups. Clinical characteristics of the *ALK*-positive group, common driver mutation group, and pan-negative group are summarized in Table 1.

**Table 1:** To compare the clinicopathologic characteristics of ALK-positive group respectively with common driver mutation group and pan-negative group.

Variable	ALK+ (n = 44)	Common Mutation (n = 411)	*p*	Pan-negative (n = 117)	*p*
	No.Samples	No.Samples		No.Samples	
**Age**					
Average≤59	30	206	0.023*	62	0.83
>59	14	205		55	
Mean(Years)	56	60		59	
Median(Years)	55	60		59	
Range(Years)	22–82	27–84		36–82	
**Sex**					
Male	19	128	0.105	68	0.09
Female	25	283		49	
**Somking History(%)**					
Never smoker	31	283	0.828	46	<0.001*
smoker	13	128		71	
**Tumor Size**					
<3 cm	23	240	0.435	46	0.139
≥3 cm	21	171		71	
**Dominant histological subtype**					
Lepidic predominant	1	48	0.056	6	0.428
Acinar predominant	15	221	0.013*	45	0.609
Papillary predominant	6	74	0.469	15	0.891
Solid predominant	19	53	<0.001*	49	0.882
Micropapillary predominant	1	4	0.432	0	0.102
mucinous adenocarcinoma	2	11	0.479	2	0.303
**Other histological pattern**					
Cribriform pattern	23	20	<0.001*	34	0.006*
Signet-ring cell	13	8	<0.001*	24	0.225
Hepatoid appearance	11	30	<0.001*	18	0.157
Extracelluar mucus	22	52	<0.001*	25	<0.001*
Any type of mucous cells	26	63	<0.001*	30	<0.001*
**Grade**					
Grade I	1	48	0.59	6	0.344
Grade II	14	294		58	
Grade III	29	69		53	
**Pleural Invasion**					
Negative	24	268	0.161	79	0.126
Positvie	20	143		38	
**Lymph Node Status**					
Negative	20	245	0.07	50	0.798
Positvie	24	166		67	
**Stage**					
I	21	221	0.445	43	0.205
II–IV	23	190		74	

* marks parameters showing statistical significance by univariate analysis.

### Pathological findings of *ALK*-rearranged lung cancers

**Figure 1:**
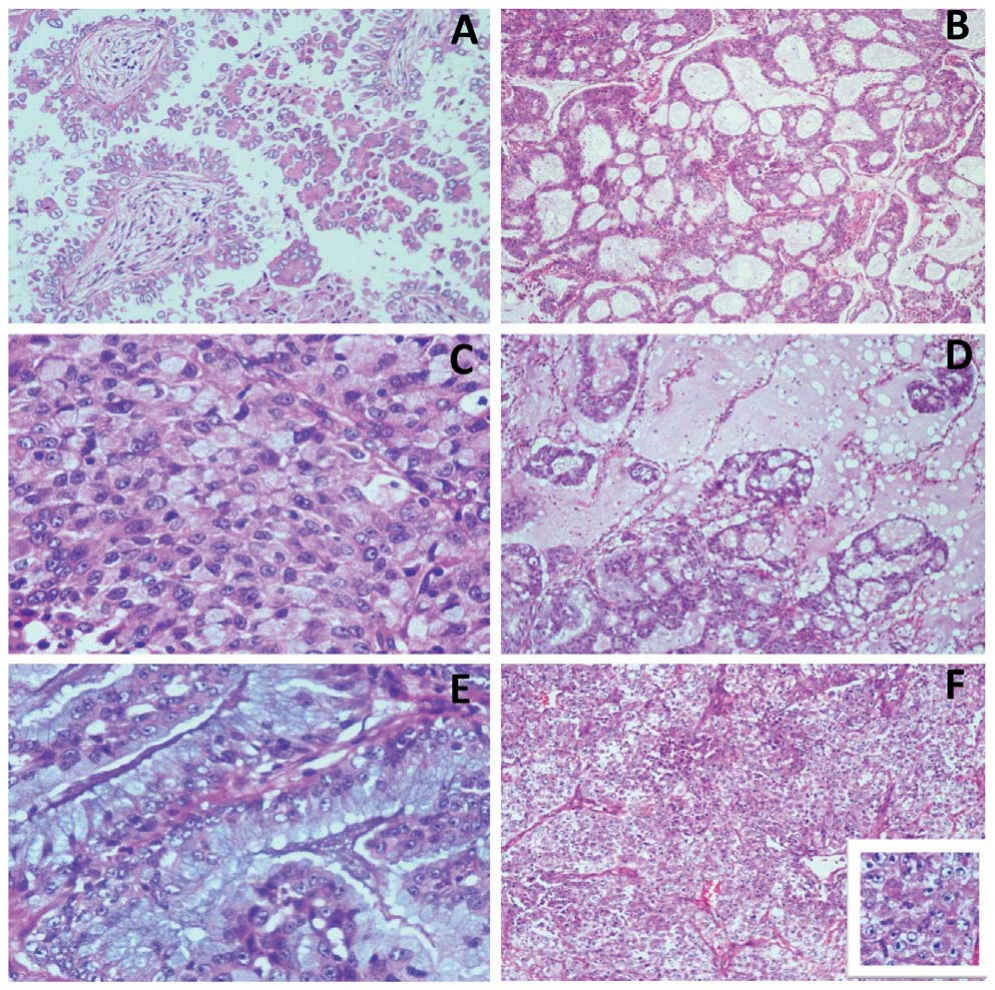
Morphology of *ALK*-rearrangement lung adnenocarcinoma. (A) papillary and micropapillary patterns; (B) mucinous cribriform pattern consisting of abundant extracellular mucus and cribriform structures; (C) solid pattern with signet ring cells; (D) mucinous cribriform pattern often floating within mucus-filled alveolar spaces; (E) mucous cells in form of goblet cells; (F) solid pattern with hepatoid tumor cells having abundant eosinophilic cytoplasm, round nuclei, and prominent nucleoli; the tumor cell nuclei are relatively monomorphic.

Table 1 summarizes the pathological features of all 44 cases. All 44 were adenocarcinomas and 2 had additional focal squamous components (account for 10% of tumor volume). According to pattern-based grading system, there were no differences in tumor grade between *ALK*-positive, common driver mutation or pan-negative groups (*p* = 0.59 and *p* = 0.344, respectively). According to the predominance subtypes based on the IASLC/ATS/ERS classification, our *ALK*-positive group had various predominant subtypes: acinar 15 cases (34.09%), solid 19 cases (43.18%), lepidic 1 case (2.27%), papillary 6 cases (13.64%), micropapillary 1 case (2.27%, Figure 1A), mucinous 2 cases (4.55%). The *ALK*-positive tumors were significantly associated with solid- and acinar- predominant patterns compared with common driver mutant adenocarcinomas (*p*<0.0001 and *p* = 0.013, respectively), while not significantly associated with other predominant histologic patterns. In addition, *ALK*-positive tumors were not significantly associated with any predominant histologic patterns compared with pan-negative adenocarcinomas.

*ALK*-positive tumors were also associated with distinct morphological features. Of the 44 tumors, 52.27% (23/44) showed a cribriform pattern (Figure 1B), either focal or diffuse. In contrast, only 20 of 411 common driver mutant (*p*<0.001) and 34 of 117 pan-negative tumors had a cribriform pattern (*p* = 0.006). There were 29.55% (13/44) of *ALK*-positive but only 2% (8/411) of common driver mutant tumors (*p*<0.001) which had at least some degree of signet ring cells (Figure 1C). Signet-ring cells were associated with a solid predominant pattern in 11 *ALK*-positive tumors, an acinar pattern in 1, and both acinar and solid patterns in one. The ratio of signet-ring cells to total tumor cells varied, with a range of 5 to 80 percent. Interestingly, *ALK*-positive tumors were not significantly associated with signet-ring cells pattern compared with pan-negative tumors, demonstrating that the signet-ring pattern could be observed in other rare oncogenic mutant lung cancer. Also found were prominent extracellular mucus and any type of mucous cells were all significantly more common in *ALK*-positive tumors (P<0.0001) compared with the other 2 group (*p*<0.001). In 22 cases (50%) there was prominent extracellular mucus, with mucus expanding into alveolar spaces at the tumor periphery (Figure 1D). Any type of mucous cells was found in 26 cases (59.09%) and had various pattern, but they were found most often in a solid or acinar pattern. Mucous cells were most often signet-ring and columnar goblet types (Figure 1E). In 2 mucinous cases, goblet cells proliferated in acinar and lepidic patterns. In 11 (25%) cases there was at least a focal population of hepatoid tumor cells with abundant eosinophilic cytoplasm, round nuclei, and prominent nucleoli in a predominantly solid pattern (Figure 1F). In addition, no single histologic pattern was completely sensitive or specific to predict *ALK* rearrangement, and even cases with signet-ring cell or cribriform patterns could be present in the other 2 groups. In all *ALK*-positive cases, the tumor cell nuclei were relatively monomorphic (Figure 1F), except for two cases showing focal areas of pleomorphism. Of the 44, 40 were TTF-1 positive, while in 6, the foci of adenocarcinoma co-expressed TTF-1 and p63.

### EML4-ALK fusion variant detection by multiplex RT-PCR and sequencing in frozen material

We investigated the presence of the *EML4-ALK* fusion variant in 161 cases which did not harbor *EGFR*, *KRAS* and *BRAF* by RT-PCR. We identified 38 cases of *EML4-ALK* fusion variant by RT-PCR. *EML4-ALK* variant 1 was detected in 19 samples. Five cases were variant 2, 7 cases were variant 3a/3b and 1 was V3b, E17; A20, E17 ins 65; A20, E17b ins 39; A20 E10a/b, E13; A20, E6a/b ins 18; A20 and E6a; A19 each (Table 2). Fusions were confirmed by direct sequencing.

**Table 2:** Comparison of RT-PCR, IHC and FISH for detection of EML4-ALK positive lung cancers.

RT-PCT	FISH			D5F3 IHC				ALK1 IHC			
	split signals (n = 38)	loss of one signal (n = 6)	overlap signals (n = 117)	0 (n = 120)	1+ (n = 3)	2+ (n = 4)	3+ (n = 34)	0 (n = 129)	1+ (n = 14)	2+ (n = 14)	3+ (n = 4)
V1(n = 19)	17	2	0	0	1	2	16	6	5	5	3
V2(n = 5)	5	0	0	0	0	0	5	2	2	2	0
V3a/3b(n = 7)	7	0	0	1	1	0	5	3	1	1	1
V3b(n = 1)	0	1	0	0	0	0	1	0	0	1	0
E17;A20(n = 1)	1	0	0	0	0	0	1	0	0	1	0
E17 ins 65;A20(n = 1)	1	0	0	0	0	0	1	0	0	1	0
E17b ins 39;A20(n = 1)	1	0	0	1	0	0	0	1	0	0	0
E10a/b, E13;A20(n = 1)	1	0	0	0	0	1	0	0	1	0	0
E6a/b ins 18;A20(n = 1)	1	0	0	0	0	0	1	0	0	1	0
E6a;A19(n = 1)	1	0	0	0	0	0	1	0	0	1	0
Negative (n = 123)	3	3	117	118	1	1	3	117	5	1	0

Abbreviation: RT-PCR: Reverse Transcription-Polymerase Chain Reaction; IHC: Immunohistochemistry; FISH: Fluorescence In Situ Hybridization; split signals: any split in the green and orange signals; loss of one signal: loss of one green signal; overlap signals: overlap of red and green signals.

### *ALK* rearrangements detection by FISH

**Figure 2:**
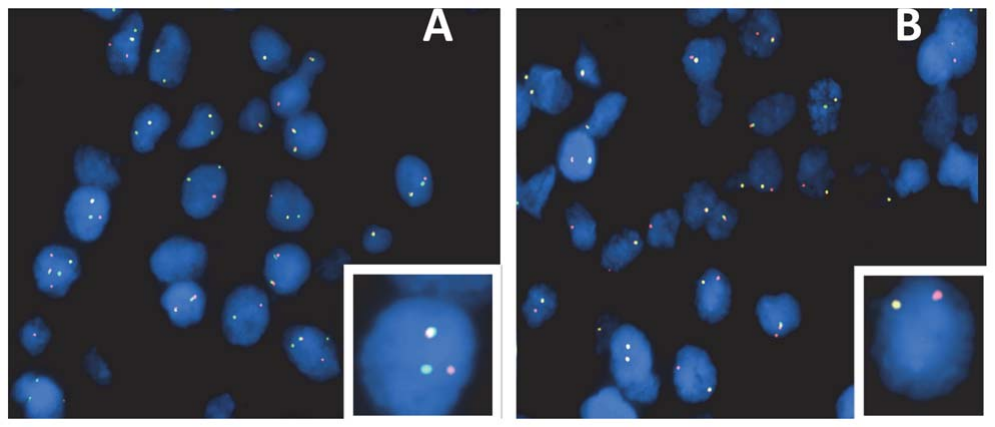
Two patterns of *ALK* gene alteration detected by FISH using Vysis LSI ALK Dual Color breakpoint probe. (A) Two distinct red and green signals. (B) Isolated red signal.

There were 161 cases were subjected to ALK FISH. ALK rearrangement is usually defined by split green and orange signals located at least twice the times of largest signal size. There were 44 cases positive as identified by FISH. Typically, two distinct splits in red and green signals were noted 38 cases (Table 2, Figure 2A). Six of the FISH-positive cases were characterized by loss of one green signal (Figure 2B). Three of these 6 cases were two variant 1 cases, one variant 3b case and three negative cases. Six RT-PCR- negative cases were positive by FISH. Two cases of the coexistence of both adenocarcinoma and focal squamous cell carcinoma demonstrated FISH-positive signal to be present only in the adenocarcinoma component.

### ALK rearrangements detection by IHC using ALK1 and D5F3 antibody

**Figure 3:**
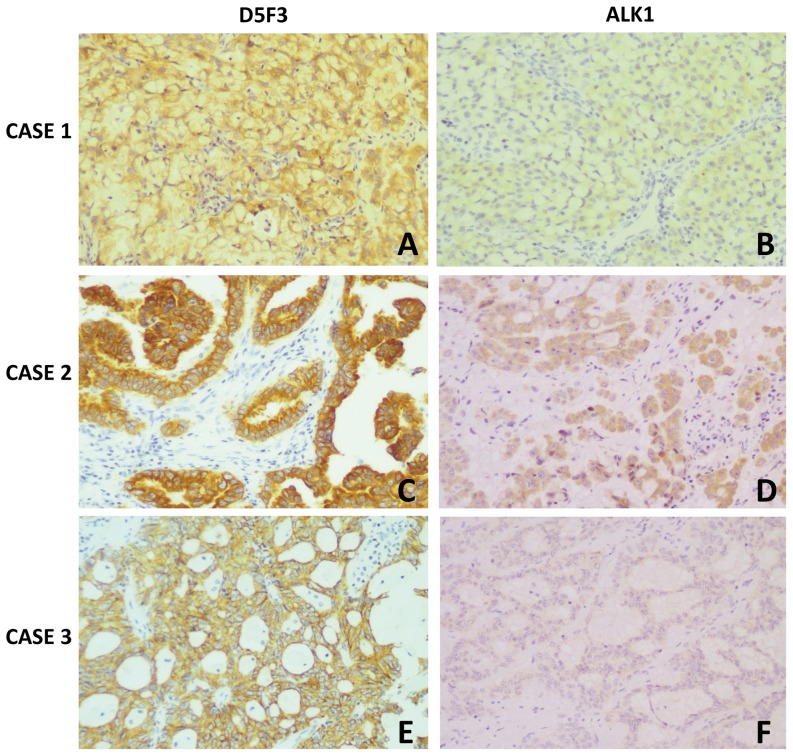
Immunohistochemical staining with D5F3 and ALK1 detects ALK-rearrangement tumors. Case 1: (A) score 3+ showing intense granular cytoplasmic staining with D5F3 antibody; (B) score 1+ showing faint cytoplasmic staining with ALK1; Case 2: (C) score 3+ showing intense granular cytoplasmic staining with D5F3; (D) score 2+ showing moderate, granular cytoplasmic staining with ALK1; Case 3: (E) score 3+ showing intense granular cytoplasmic staining with D5F3; (F) score 1+ showing faint, barely discernible cytoplasmic staining with ALK1.

ALK IHC testing in lung cancer remains challenging because of the relatively low expression of ALK protein, with many results showing this to be the case using ALK1 antibody. To determine whether IHC using the D5F3 antibody might be better, we evaluated both in 161 cases. ALK1 antibody expression was detected in 32, resulting in IHC scores of 1 (n = 14), 2 (n = 14), and 3 (n = 4), whereas 28 patients were FISH-positive (Table 2). We observed that all IHC 0 cases were FISH-negative, all IHC 3+ and 2+ cases were FISH-positive, 4 IHC 1+ cases were FISH-negative, and 10 IHC 1+ cases were FISH-positive. Among the 32 ALK1 IHC-positive cases, only 4 IHC 3+ exhibited a strong cytoplasmic staining intensity, observed in 20% to 100% of positive cells (mean score of 60%). IHC1+ and 2+ cases were only observed in 10% to 50% of positive cells (mean score of 30%) (Figures 3B, 3D, 3F).

In our study, the D5F3 IHC test showed very high sensitivity and specificity for detecting ALK expression. D5F3 immunopositivity was identified in 41 cases (Figures 3A, 3C, 3E). Positive immunostaining was found only in the adenocarcinoma component of the 2 adenosquamous carcinomas. Among 41 D5F3-positive cases, 34 were 3+ exhibiting strong cytoplasmic staining intensity, observed in 60% to 100% of positive cells (mean score of 80%). Of the remaining 7 cases, 2 cases exhibited cytoplasmic and nuclear positive staining. There were 4 IHC-positive that were 2+, showing moderate cytoplasmic staining, and 3 IHC-positive cases were 1+, showing faint cytoplasmic staining. No background was observed in stromal cells or within normal lung. Compared the immunoactivity of ALK1 antibody, D5F3 showed more intense staining of tumor cells. The relative amount of staining area with D5F3 was also much higher. Therefore, intensity scores of D5F3 antibody staining were higher than ALK1 antibody (Table 2). Interestingly, D5F3 IHC results in 1 case illustrate that heterogeneous staining can occur. One tissue block showed no staining despite repeated IHC testing, while another block showed positive staining. FISH analysis confirmed the presence of the genetic abnormality. We considered the possibility that the differing results was due to heterogeneity.

### Comparison between IHC and RT-PCR

There were 26 of 38 cases that were RT-PCR-positive were ALK1 IHC-positive, with the other 12 cases being IHC negative. Sensitivity of ALK1 IHC for the detection of ALK rearrangement was thus 68.4% (26 of 38) when compared with RT-PCR. However, 36 of 38 RT-PCR-positive cases were D5F3-positive (Table 3). Thus, sensitivity of D5F3 was 94.7% (36 of 38) when compared with RT-PCR (Table 3). D5F3-positive cases included variant 1, 2, 3a/3b, 3b, E17; A20, E17 ins 65; A20, E17b ins 39; A20, E10a/b, E13; A20, E6a/b ins 18; A20 and E6a;A19. Two D5F3-negative cases of the 38 RT-PCR-positive cases were a variant 3a/b and a E17b ins 39; A20 case (Table 2). Five of 123 RT-PCR negative cases were D5F3-positive.

**Table 3:** Comparison between RT-PCR and IHC.

IHC		RT-PCR	
		Positive	Negative
D5F3	Positive	36	5
	Negative	2	118
ALK1	Positive	26	2
	Negative	12	121

Abbreviation: RT-PCR: Reverse Transcription-Polymerase Chain Reaction; IHC: immunohistochemistry.

### Comparison between IHC and FISH

There were 28 of 44 FISH-positive cases that were ALK1 IHC-positive, and 4 ALK1 IHC-positive cases that were FISH-negative. The overall sensitivity and specificity of the ALK1 IHC in comparison with FISH were 64% and 91%, respectively. In contrast, 40 of 44 FISH-positive cases were D5F3-positive. Four of 44 FISH-positive cases were D5F3-negative. One FISH negative case was D5F3-positive. Overall sensitivity and specificity of D5F3 in comparison with FISH were 91% and 98% (Table 4).

**Table 4:** Comparison between FISH and IHC.

IHC		FISH	
		Positive	Negative
D5F3	Positive	40	1
	Negative	4	116
ALK1	Positive	28	4
	Negative	16	113

Abbreviation: FISH: Fluorescence In Situ Hybridization; IHC: immunohistochemistry.

## Discussion

Although *ALK*-rearranged lung cancers make up only 5% to 7% of all NSCLC cases, they have demonstrated an impressive responsiveness to the ALK tyrosine kinase inhibitor, crizotinib, and represent a therapeutically important subcategory. Previous studies have evaluated ALK rearrangement lung cancer, but the comprehensive analysis of *ALK*-rearranged lung cancers in Chinese has not well assessed. We identified 44 cases of *ALK*-rearranged cases in 572 lung cancers. In our series, these tumors lacked mutation of *EGFR, KRAS or BRAF*, in agreement with the prior observations [14]. In previous reports, results of detailed clinicopathologic analyses of *ALK*-rearranged lung cancers have varied. A thorough study of clinicopathologic features of such patients compared with the common mutations *EGFR* and *KRAS* or rare unknown mutations is an emerging issue with clinical interest. We sought to compare the clincopathologic features of *ALK*-rearranged lung cancer with the common driver mutations and pan-negative tumors.

Our main clinic findings of our study were as follows: (1) *ALK*-rearranged lung cancers were more frequent in younger patients compared with the common drive mutation group (*p* = 0.023), in agreement with previous studies [4], [15], while there was no significance difference compared with patients in pan-negative group (*p* = 0.83). (2) *ALK*-rearranged patients associated with no smoking history compared with the pan-negative group (*p*<0.001), while smoking history of *ALK*-rearranged patients was not different from the common drive mutation group (*p* = 0.828). (3) There were no differences in sex, tumor size, lymph node status, tumor grade or stage between *ALK*-positive, common driver mutation or pan-negative groups. This discrepancy may be due to the small number of cases examined in other study, or different experiment grouping design, or differences in the ethnicity. Therefore, the clinical characteristics alone are not sufficient to selecting individual cases for further testing for *ALK* rearrangement.

In our study, all 44 *ALK*-rearranged lung cancers were adenocarcinomas, with 2 cases having additional focal squamous components. Interestingly, in neither case was the *ALK* rearrangement present in the squamous cell component, confirmed by both FISH and IHC. This is in contrast with one report where *ALK* rearrangement coexisted in both components [15]. Although most *ALK*-rearranged lung cancers are adenocarcinomas, other histological types should not exclude its presence. In our study, *ALK*-rearranged tumors were characterized by a solid or acinar growth pattern and lack of lepidic growth. When compared with common driver mutation tumors, solid or acinar-predominant pattern, cribriform structure, signet-ring cells, hepatoid appearance, prominent extracellular mucus, and any type of mucous cells were all significantly associated with the *ALK*-positive tumors. This is in agreement with a previous report [13]. When compared with pan-negative tumors, only cribriform structure, prominent extracellular mucus and any type of mucous cells were significantly associated with *ALK*-positive lung adenocarcinomas. Therefore, our data showed cribriform structure, prominent extracellular mucus and any type of mucous cells pattern are sensitive and/or specific to predict *ALK* rearrangement. In most *ALK-*rearranged tumors, tumor nuclei were relatively monomorphic, except for 2 cases showing focal areas of pleomorphism. These distinct morphologic features of *ALK* rearrangement tumors are in accordance with previous reports from both Western and Asian cohorts [15], [16] and may be helpful in selecting cases for further accurate molecular testing.

Although p63 positivity is often used as a marker of squamous cell carcinoma in diagnostic pathology, we found that a few *ALK*-rearranged tumors coexpressed p63 and TTF1 in the adenocarcinoma component. Our data are much different from a previous report which demonstrated that more than half of their tumors co-expressed TTF-1 and p63, showing diffuse staining of both [16]. If p63 was used as a squamous cell marker to make a pathological diagnosis, *ALK*-rearranged tumors could be misdiagnosed as adenosquamous cell carcinomas.

Accurate identification of *ALK*-rearranged tumors requires a sensitive and specific testing method. Currently, several such methods exist, such as IHC, FISH, and RT-PCR. RT-PCR is suited to detect *EML4-ALK* or alternative fusion transcripts [14], but is not available in many routine pathology departments. In addition, the majority of specimens are stored as FFPE, in which case RNA may be substantially degraded. Furthermore, RT-PCR may not detect all the translocations involving the gene. FISH, using the ALK break-apart probe, was the standard test for enrollment in a clinical trial with crizotinib [6]. However, FISH is considered low-throughput, so is not suitable for detecting *ALK* rearrangements in large-scale screening. IHC, however, is a low-cost, relatively easy technique for screening and diagnosis *ALK*-rearranged tumors in routine surgical pathology practice, although, some ALK antibodies have been reported to lack specificity and sensitivity [4].

We evaluated *EML4-ALK* fusion variants using RT-PCR and identified that E13; A20 (variant 1), E6a/b; A20 (variant 3a/b), E20; A20 (variant 2) are the most common variants. This frequency of distribution is quite similar to those reported [17]. In our study, 6 RT-PCR-negative cases were FISH-positive, suggesting the present of other unknown variants. In our study, D5F3 antibody showed very little nonspecific or background immunostaining. Its sensitivity and specificity (91% and 98%, respectively) is much higher than that (63.6% and 93%) of ALK1 IHC. There was only 1 false-positive D5F3 case, while 4 ALK1+ cases were false-positive, and confirmed by FISH. Moreover, D5F3 showed a much higher relative amount of staining and intensity than did the ALK1. ALK1 and D5F3 IHC were positive only in the adenocarcinoma component, but not in the squamous components in 2 adenosquamous carcinomas. Alternatively, D5F3 is simply better than the ALK1. Therefore, D5F3 may be the optimal screening tool for detecting *ALK* rearrangements, and could be routinely applicable in clinical practice. Our finding is in line with another work [7], which demonstrated that D5F3 had excellent sensitivity and specificity (100% and 99%, respectively). Similarly, it has been demonstrated that D5F3 (Cell Signaling by ADVANCE) showed the greatest combination of sensitivity (100%) and specificity (75%) and produced no false-negative results [17].

In conclusion, we identified 44 *ALK*-rearranged lung adenocarcinomas, the largest reports in China to date. In our study, *ALK*-rearranged lung adenocarcinomas were associated with characteristic morphology such as cribriform structure, prominent extracellular mucus, and any type of mucous cell pattern. FISH remains a highly useful and accurate method to identify *ALK* rearrangements while RT-PCR may not be able to detect all translocations and is technically limited in many pathology departments. IHC, although not as sensitive as FISH, remains the preferred technique for such cases. Also, D5F3 could be the optimal screening tool for detecting *ALK* rearrangements.
